# A Review of the Author’s Method of Anchoring the Kidneys*Read before the Ohio State Medical Society at the meeting Held at Columbus, May 27, 28, and 29, 1896.

**Published:** 1896-12

**Authors:** Harvey Reed

**Affiliations:** Columbus, Ohio; Professor of the Principles and Practice of Surgery and Clinical Surgery, Ohio Medical University; Surgeon to the Protestant and University Hospitals


### A REVIEW OF THE AUTHOR'S METHOD OF ANCHORING THE KIDNEY.*

R. HARVEY REED, M. D., COLUMBUS, OHIO.

Professor of the Principles and Practice of Surgery and Clinical Surgery, Ohio Medical University; Surgeon to the Protestant and University Hospitals.

The frequency with which surgeons meet both floating and movable kidney has long since attracted attention as to the best method of anchoring this organ so as to preserve its normal functions. The multitude of complex disturbances and reflex symptoms associated with a floating or movable kidney, are such that the surgeon is constantly called upon to render relief. These abnormal conditions may last for years without serious results, yet they are liable to give rise to degenerative changes which may necessitate a nephrectomy or a nephrotomy at any moment. Palliative treatment, by means of rest and bandaging, as a rule, avails but little. The difficulty of holding a kidney in place with a bandage, is such that little reliance can be placed on this method of treatment.

From the fact that this abnormal condition is chiefly a source of annoyance rather than danger, patients hesitate in submitting to an operation for the purpose of anchoring the kidney, as it seems to them like a very large undertaking for the purpose of accomplishing very small results. It is hard to make them understand the importance of having the kidney anchored and the danger that is likely to arise from neglect of the proper surgical treatment. At the same time we can hardly blame them or their family physician for not urging an operation which requires a large oblique gash through the lumbar muscles and a number of buried sutures which are difficult to insert. Only those who have attempted to perform this operation can appreciate how hard it is to hold the kidney in place by the old-fashioned method until it is sutured to the deep muscles of the back. The difficulty of this procedure stimulated me to devise a nw operation which had for its object, simplicity, rapidity, and efficiency.

Referring to a paper read before the Columbus Academy of Medicine, November 19, 1894, on "A New Method of Anchoring the Kidney," published in the *Columbus Medical Journal*,

*Read before the Ohio State Medical Society at the meeting held at Columbus, May 27, 28, and 29, 1896.

December 25, 1894, you will find that my operation consists "in making the ordinary perpendicular abdominal incision over the median line of the kidney. As a rule, it need not exceed two and a half inches in length, depending largely on the thickness of the abdominal walls. Having made the incision sufficiently large to get the fingers in and bring the kidney to its normal place, I then use a long needle which I have had made on purpose, seven inches in length. Two of these needles are threaded with aseptic silkworm gut or aseptic silk, using but one ligature. Having placed the kidney in its normal position (and in the case of a floating kidney scored the peritoneum so as to favor adhesions), I now insert my first needle through the upper and inner part of the cortical substance of the kidney directly through the muscles of the back, bringing it out between the eleventh and twelfth ribs. The second needle, which is on the other end of the ligature, is also passed through in a similar manner, about an inch from its fellow, through the upper and outer cortical substance of the kidney, making, as you will recognize, a staple stitch. These ligatures are tied on the integument of the patient's back by an assistant. If necessary, another suture is inserted in a similar manner through the outer margin of the kidney, the first needle of the second suture being passed about an inch below the last needle of the first suture, and the second needle of the second suture about an inch below the first needle of the second suture, through the cortical substance of the outer portion of the kidney."

You will readily see that this is a very simple operation, does not involve any vital structures, and can be performed in a few moments, with little or no danger to the patient, while the results have been even more than anticipated. In explaining the method I had adopted to my friends, I found but practically one criticism, and that was a lack of confidence in obtaining satisfactory results. Recognizing the fact that it required several sutures, by the old method, to hold the kidney in place, they did not see how it was possible for one or two sutures to accomplish the same. If you stop to study the difference between the two methods, you will readily observe that the new method "clinches," so to speak, the kidney by a staple suture, while the old method simply sutured the posterior portion to the deep lumbar muscles. The merest tyro will readily see the mechanical difference between the two sutures. The one not only embraces the entire kidney, but pierces the lumbar muscles and is reinforced by the integument on the back, while the other simply involves a portion of the friable cortex of the kidney and a small portion of the tenderloin; hence it is quite evident that more sutures would be required by the old method than by the new.

Since devising this plan for anchoring the kidney, I have had an opportunity for demonstrating its practical utility in five* cases operated by myself and one by my colleague, Dr. Means, with the most satisfactory results in each case. The rapidity with which the operations were done is one of the marked features. It is only necessary to make a very small opening into the abdominal cavity, bring the kidney to its normal position, pierce it with the needles as above described, tie the sutures over a piece of iodoform gauze on the back, and close the abdominal wound. There are seldom any constitutional symptoms following the operation. The patient has little or no pain or rise of temperature, while the pulse remains practically normal. In about ten days the suture can be removed, leaving the kidney entirely free from any foreign substance. I usually have the patient remain quiet from two to three weeks after the operation.

Up to date there has not been a single instance of a return of the disease, so far as I have any knowledge, the patients are all enjoying good health, and are entirely free from the reflex symptoms which were so annoying prior to the operation. In two of these cases it was my fortune to have an opportunity to examine the result; in one case several months afterwards, and the other nearly two years. In each case the patient had to be operated for ovarian trouble, and in each I made a careful examination of the kidney which had been anchored, and found it firmly fixed, and, so far as I was able to judge, in a perfectly healthy condition.

I do not claim that the few cases which I have reported are sufficient to establish the fact that this method is without fault, but I do claim that up to date the results secured are better than those usually obtained by other methods.

---

*The author operated his sixth case, at the University Hospital, during the recent meeting of the Ohio State Medical Society, which made a prompt and uneventful recovery, making a total of seven cases with seven recoveries by this method.

